# Analysis of risk factors associated with intimal hyperplasia in arteriovenous fistulas among patients undergoing hemodialysis

**DOI:** 10.1097/MD.0000000000049682

**Published:** 2026-07-17

**Authors:** Mingli Liang, Xiaoli Liao

**Affiliations:** a Nephrology Rheumatology and Immunology Department, Mengcheng County First People’s Hospital, Bozhou City, Anhui Province, China; b College of Medical Technology and Nursing, Hunan Institute of Transportation Engineering, Hengyang City, Hunan Province, China.

**Keywords:** albumin, arteriovenous fistula, C-reactive protein, diabetes mellitus, hemodialysis, intimal hyperplasia

## Abstract

Arteriovenous fistula (AVF) intimal hyperplasia (IH) is a major pathological basis of AVF stenosis and dysfunction in patients undergoing maintenance hemodialysis. This single-center retrospective observational cohort study enrolled adult hemodialysis patients with a native AVF at our institution between May 2021 and May 2023. IH was determined primarily by duplex ultrasound (DUS) evidence of pathologic venous intimal thickening and/or hemodynamically significant stenosis attributable to IH, with angiographic confirmation when clinically indicated. Demographic characteristics, hemodialysis vintage, dialysis prescription and adequacy, vascular access profiles, comorbidities, medication exposure, and routine laboratory indices were extracted from electronic medical records, the hemodialysis information platform, and the vascular access imaging database. Comparisons were performed between the IH group (n = 38) and the control group (n = 126), followed by univariate and multivariable logistic regression analyses. Patients with IH were slightly older and had longer hemodialysis vintage and a longer interval from AVF creation to evaluation. The IH group also showed higher rates of diabetes mellitus and recent infection, higher inflammatory burden reflected by C-reactive protein (CRP) and neutrophil-to-lymphocyte ratio (NLR), lower albumin, higher d-dimer, and higher serum phosphate. In multivariable analysis, longer time from AVF creation to evaluation, diabetes mellitus, higher CRP, and higher phosphate independently correlated with IH, whereas higher albumin was protective. These findings suggest that cumulative access exposure, metabolic disease, systemic inflammation, nutritional status, and disordered mineral metabolism jointly contribute to IH risk and may inform targeted surveillance and preventive management in routine hemodialysis care.

## 1. Introduction

Arteriovenous fistulas (AVFs) remain the preferred vascular access for maintenance hemodialysis because of their favorable long-term performance relative to other access options; yet, both early maturation failure and late dysfunction continue to impose substantial clinical and economic burdens. Contemporary educational updates and reviews emphasize that access-related complications remain common across real-world dialysis programs and require systematic clinical monitoring and timely intervention.^[1]^ Among the pathological substrates of AVF dysfunction, venous intimal hyperplasia (IH) is widely recognized as the dominant lesion underlying progressive stenosis, particularly within the anastomotic and juxta-anastomotic regions and the outflow vein. Mechanistic syntheses in recent years highlight that IH reflects an integrated response to surgical and cannulation injury, disturbed shear stress, endothelial dysfunction, inflammatory cell recruitment, and proliferation/migration of smooth muscle cells and myofibroblasts with extracellular matrix remodeling.^[2,3]^ The heterogeneity of IH development suggests that risk is shaped by both local hemodynamic factors and systemic patient-level conditions. Recent translational studies have expanded the mechanistic landscape beyond classical “injury-flow” paradigms to include metabolic reprogramming and immune-inflammatory signaling networks that may predispose to stenotic remodeling.^[4]^ Parallel clinical research has increasingly adopted modern modeling approaches to improve risk stratification for AVF patency outcomes, including nomograms and interpretable machine learning frameworks, underscoring the value of integrating routine clinical and laboratory variables into predictive tools.^[5]^

Diabetes mellitus represents one of the most clinically relevant systemic exposures in this context. Emerging omics-based and clinical investigations support a close association between hyperglycemia-related vascular injury and AVF stenosis, potentially through oxidative stress, advanced glycation end products, impaired endothelial repair, and heightened pro-inflammatory signaling.^[6]^ In addition, the inflammatory milieu of end-stage kidney disease has been increasingly operationalized using composite or index-based approaches. Recent prediction-model studies focusing on inflammation-derived markers reinforce the concept that systemic inflammatory activation is not only relevant to AVF non-maturation but may also contribute to later stenotic phenotypes linked to IH.^[7]^ Disordered mineral metabolism may provide an additional mechanistic bridge between systemic disease and access remodeling. Recent cohort evidence suggests that hyperphosphatemia is associated with AVF dysfunction, supporting the biological plausibility that phosphate-driven endothelial injury, vascular stiffness, and pro-calcific signaling could facilitate IH-prone remodeling in susceptible patients.^[8]^ Finally, evolving diagnostic frameworks and lesion classifications, together with increasing reliance on duplex ultrasound for structured assessment, indicate a need for clinically grounded risk-factor analyses that align with contemporary definitions of hemodynamically significant access lesions.^[9,10]^

Against this background, a focused evaluation of demographic characteristics, dialysis-related parameters, vascular access profiles, comorbidities, medication exposures, and routinely available laboratory indices may help clarify the independent correlates of IH in AVFs among hemodialysis patients. Such evidence can support more individualized surveillance strategies and inform preventive management targeted at modifiable systemic contributors to stenotic remodeling.

## 2. Materials and methods

### 2.1. Study design

This was a single-center retrospective observational cohort study based on routinely collected clinical and imaging data. This study was approved by the Ethics Committee of Mengcheng County First People’s Hospital.This single-center retrospective observational cohort study enrolled adult patients undergoing maintenance hemodialysis at our institution between May 2021 and May 2023 who had a native arteriovenous fistula (AVF) created and/or followed up during the study period. Eligible patients were required to have complete baseline demographic and clinical data, documented AVF-related assessments, and objective evaluation for AVF lesions allowing determination of the presence of intimal hyperplasia. Patients were excluded if they were younger than 18 years; had non-native access or lacked a functioning AVF; had a history of prior AVF revision before index evaluation that could confound lesion attribution; had incomplete key clinical, laboratory, or imaging records; had acute systemic infection at the time of assessment; or had other major conditions likely to substantially influence vascular remodeling or short-term survival. According to whether intimal hyperplasia of the AVF was identified during follow-up, patients were assigned to the intimal hyperplasia group or the control group. The study was conducted in accordance with the Declaration of Helsinki and was approved by the institutional medical ethics committee, and written informed consent was obtained from all participants.

### 2.2. Diagnostic criteria for intimal hyperplasia in native AVFs

In this study, intimal hyperplasia (IH) of native arteriovenous fistulas was diagnosed primarily based on imaging evidence of pathologic intimal thickening of the draining vein and/or hemodynamically significant AVF stenosis attributable to IH. Duplex ultrasound (DUS) served as the first-line modality, with IH suggested by circumferential, hypoechogenic parietal thickening on B-mode/CFI (color flow imaging) in the outflow vein, consistent with standardized DUS descriptions of AV access lesions.^[11]^ For operational definability, IH could be defined as venous intima-media thickness (IMT) ≥ 0.4 mm, with IMT ≥ 0.6 mm indicating more prominent IH in line with a recent DUS-based classification system for native AVFs.^[9]^ Hemodynamically significant stenosis was considered present when DUS demonstrated ≥ 50% luminal diameter reduction compared with an adjacent normal segment, together with focal velocity acceleration, and supportive Doppler criteria included a peak systolic velocity (PSV) ≥ 500 cm/s predicting ≥ 50% stenosis in AVFs and/or a PSV ratio > 3 with an absolute PSV > 400 cm/s at the anastomosis.^[12]^ When clinically indicated, angiography was used to confirm lesion severity and location. In addition, reduced access flow on DUS (e.g., <500 mL/min) was interpreted as a supportive functional marker of clinically relevant outflow compromise associated with stenotic pathology, including IH. All patients underwent protocolized scheduled DUS surveillance at predefined intervals. DUS examinations were performed according to routine clinical practice, including evaluation for access dysfunction, abnormal dialysis parameters, difficult cannulation, reduced access flow, or physician-directed surveillance assessment.

### 2.3. Data collection

Clinical data were extracted retrospectively from the hospital electronic medical record system, the hemodialysis information platform, and the vascular access imaging database. A standardized case report form was used to collect baseline demographic and disease-related variables, including age, sex, body mass index, primary kidney disease, and hemodialysis vintage. Dialysis prescription and treatment parameters were recorded for the index period, including dialysis frequency, session duration, anticoagulation regimen (unfractionated heparin, low-molecular-weight heparin, or other strategies), and routinely reported dialysis adequacy indices (*Kt*/*V* and urea reduction ratio). Vascular access characteristics were obtained from operative notes and subsequent access records, covering AVF location (wrist vs elbow), access configuration (radiocephalic vs brachiocephalic), time from AVF creation to the index evaluation, and prior access-related adverse events (history of thrombosis and/or stenosis-related episodes requiring clinical assessment or intervention). Time from AVF creation to the index evaluation was analyzed primarily as a marker of cumulative AVF exposure duration and follow-up time rather than a direct causal variable, acknowledging the possibility of surveillance-related bias inherent to retrospective observational assessment. Comorbidities were identified using documented physician diagnoses and discharge codes, including hypertension, diabetes mellitus, coronary artery disease, heart failure, cerebrovascular disease, and peripheral artery disease or clinical indicators suggestive of vascular calcification. Infection-related variables were recorded based on documented infectious events within 6 months prior to the index evaluation. Medication exposures were captured from prescription records during the same index period, including statins, antiplatelet agents, renin–angiotensin system inhibitors, and chronic kidney disease–mineral and bone disorder therapies (phosphate binders, active vitamin D analogs, and calcimimetics).

Laboratory parameters were collected from routine blood tests closest to the index imaging assessment, using a prespecified window when feasible. Inflammatory indices included C-reactive protein, white blood cell count, and the neutrophil-to-lymphocyte ratio. Nutritional and protein metabolism markers included albumin and total protein. Anemia and iron-related variables included hemoglobin, ferritin, and transferrin saturation. Coagulation-related indices included platelet count and d-dimer. Uremia-related markers included blood urea nitrogen and serum creatinine. Mineral metabolism variables included serum calcium, phosphate, intact parathyroid hormone, and alkaline phosphatase. IH status was determined from vascular access duplex ultrasound reports and, when available, angiographic confirmation, based on documented morphological and hemodynamic evidence of IH-related stenotic lesions in the anastomotic or outflow venous segments. Data extraction was performed independently by 2 investigators, with discrepancies resolved by consensus review against the original records to ensure accuracy and completeness of the analytic dataset.

### 2.4. Statistical analysis

All statistical analyses were performed using IBM SPSS Statistics, version 26.0 (IBM Corp., Armonk). Continuous variables are presented as mean ± standard deviation and were compared between the IH group and the control group using the independent-samples *t* test. Categorical variables are summarized as counts and percentages and were compared using the χ^2^ test. Univariate logistic regression was conducted to screen potential factors associated with AVF intimal hyperplasia. Variables with *P* < .10 in univariate analysis and/or deemed clinically relevant were entered into a multivariable logistic regression model to identify independent correlates of IH, with results reported as odds ratios (ORs) or adjusted odds ratios (aORs) with 95% confidence intervals. All tests were 2-sided, and *P* < .05 was considered statistically significant.

## 3. Results

### 3.1. Baseline demographic, dialysis prescription, and vascular access characteristics

The IH group (n = 38) and the control group (n = 126) were compared across demographic variables, dialysis prescription parameters, and vascular access characteristics (Table 1). Patients with IH were slightly older than controls (63.8 ± 10.2 vs 60.5 ± 9.6 years) and had a longer hemodialysis vintage (5.2 ± 2.9 vs 4.3 ± 2.5 years), whereas sex distribution and BMI were comparable between groups (male: 63.2% vs 61.1%; BMI: 23.6 ± 3.1 vs 23.9 ± 3.0 kg/m^2^). The composition of primary kidney disease was similar between groups, with diabetic nephropathy accounting for 39.5% of IH cases and 34.9% of controls. Dialysis prescription patterns were largely balanced, including dialysis frequency (3 sessions/wk: 92.1% vs 93.7%) and session duration (4.02 ± 0.31 vs 3.95 ± 0.36 hours). Dialysis adequacy showed a trend toward lower *Kt*/*V* in the IH group (1.35 ± 0.22 vs 1.42 ± 0.20), while URR was modestly lower in IH patients (66.8 ± 6.5% vs 68.4 ± 6.2%). Regarding vascular access, the distribution of wrist versus elbow AVFs and radiocephalic versus brachiocephalic configurations did not differ significantly. However, the interval from AVF creation to index evaluation was longer in the IH group (24.0 ± 8.7 vs 20.2 ± 6.9 months). Prior access-related adverse events were more frequent among IH patients, with higher rates of stenosis-related history (28.9% vs 12.7%) and a borderline higher prevalence of prior thrombosis (21.1% vs 9.5%; Table 1).

**Table 1:** Baseline demographics, dialysis prescription, and vascular access characteristics between groups.

Variable	IH group (n = 38)	Control group (n = 126)	Test statistic	*P* value
Demographic characteristics				
Age, yr	63.8 ± 10.2	60.5 ± 9.6	*t* = 1.77	.082
Male sex, n (%)	24 (63.2)	77 (61.1)	χ^2^ = 0.05	.820
BMI, kg/m^2^	23.6 ± 3.1	23.9 ± 3.0	*t* = −0.53	.600
Hemodialysis vintage, yr	5.2 ± 2.9	4.3 ± 2.5	t = 1.73	.089
Primary kidney disease, n (%)			χ^2^ = 0.45	.930
Diabetic nephropathy	15 (39.5)	44 (34.9)		
Hypertensive nephrosclerosis	7 (18.4)	27 (21.4)		
Chronic glomerulonephritis	11 (28.9)	35 (27.8)		
Others	5 (13.2)	20 (15.9)		
Dialysis prescription and treatment characteristics				
Dialysis frequency, n (%)			χ^2^ = 0.11	.739
3 sessions/wk	35 (92.1)	118 (93.7)		
2 sessions/wk	3 (7.9)	8 (6.3)		
Session duration, h	4.02 ± 0.31	3.95 ± 0.36	*t* = 1.17	.245
Anticoagulation regimen, n (%)			χ^2^ = 0.30	.859
Unfractionated heparin	19 (50.0)	69 (54.8)		
Low-molecular-weight heparin	16 (42.1)	49 (38.9)		
Others	3 (7.9)	8 (6.3)		
*Kt*/*V*	1.35 ± 0.22	1.42 ± 0.20	*t* = −1.75	.085
URR, %	66.8 ± 6.5	68.4 ± 6.2	*t* = −1.34	.184
Vascular access characteristics				
AVF type/location, n (%)			χ^2^ = 1.39	.238
Wrist AVF	22 (57.9)	86 (68.3)		
Elbow AVF	16 (42.1)	40 (31.7)		
AVF configuration, n (%)			χ^2^ = 1.14	.286
Radiocephalic AVF	25 (65.8)	94 (74.6)		
Brachiocephalic AVF	13 (34.2)	32 (25.4)		
Time from AVF creation to evaluation, mo	24.0 ± 8.7	20.2 ± 6.9	*t* = 2.47	.017
History of access thrombosis, n (%)	8 (21.1)	12 (9.5)	χ^2^ = 3.62	.057
History of access stenosis/intervention indication, n (%)	11 (28.9)	16 (12.7)	χ^2^ = 5.60	.018

AVF = arteriovenous fistula, BMI = body mass index, IH = intimal hyperplasia, *Kt*/*V* = dialyzer clearance of urea × time/volume of distribution of urea, SD = standard deviation, URR = urea reduction ratio.

### 3.2. Comorbidities and medication use

Hypertension was highly prevalent in both groups (89.5% vs 86.5%) without a significant difference (χ^2^ = 0.23, *P* = .632). In contrast, diabetes mellitus was more common in the IH group than in controls (57.9% vs 34.9%; χ^2^ = 6.41, *P* = .011). Similarly, peripheral artery disease or clinical indicators suggestive of vascular calcification were observed more frequently among IH patients (28.9% vs 11.1%; χ^2^ = 7.19, *P* = .007). A history of recent infection within 6 months was also higher in the IH group (26.3% vs 9.5%; χ^2^ = 7.09, *P* = .008). The between-group differences in coronary artery disease (31.6% vs 19.0%), heart failure (15.8% vs 7.9%), and cerebrovascular disease (18.4% vs 11.1%) did not reach statistical significance in this dataset (all *P* > .05). Regarding medication exposure, antiplatelet therapy was more frequently used in the IH group (42.1% vs 23.8%; χ^2^ = 4.84, *P* = .028), whereas statin use (42.1% vs 43.7%) and RAS inhibitor therapy (52.6% vs 57.1%) were comparable between groups. Treatments related to CKD–MBD, including phosphate binders, active vitamin D analogs, and calcimimetics, did not differ significantly (all *P* > .05; Table 2).

**Table 2:** Comorbidities and medication use between groups.

Variable	IH group (n = 38)	Control group (n = 126)	Test statistic	*P* value
Major cardiovascular and metabolic comorbidities				
Hypertension, n (%)	34 (89.5)	109 (86.5)	χ^2^ = 0.23	.632
Diabetes mellitus, n (%)	22 (57.9)	44 (34.9)	χ^2^ = 6.41	.011
Coronary artery disease, n (%)	12 (31.6)	24 (19.0)	χ^2^ = 2.68	.102
Heart failure, n (%)	6 (15.8)	10 (7.9)	χ^2^ = 2.04	.153
Cerebrovascular disease, n (%)	7 (18.4)	14 (11.1)	χ^2^ = 1.40	.237
Peripheral artery disease/clinical vascular calcification signs, n (%)	11 (28.9)	14 (11.1)	χ^2^ = 7.19	.007
Inflammation-/nutrition-related background				
Recent infection within 6 mo, n (%)	10 (26.3)	12 (9.5)	χ^2^ = 7.09	.008
Chronic inflammatory condition, n (%)	5 (13.2)	9 (7.1)	χ^2^ = 1.35	.245
Key medications				
Statin therapy, n (%)	16 (42.1)	55 (43.7)	χ^2^ = 0.03	.866
Antiplatelet agents, n (%)	16 (42.1)	30 (23.8)	χ^2^ = 4.84	.028
RAS inhibitors, n (%)	20 (52.6)	72 (57.1)	χ^2^ = 0.24	.623
Phosphate binders, n (%)	28 (73.7)	84 (66.7)	χ^2^ = 0.66	.415
Active vitamin D analogs, n (%)	17 (44.7)	48 (38.1)	χ^2^ = 0.54	.463
Calcimimetics, n (%)	5 (13.2)	12 (9.5)	χ^2^ = 0.41	.519

CKD–MBD = chronic kidney disease–mineral and bone disorder, IH = intimal hyperplasia, RAS = renin–angiotensin system.

### 3.3. Laboratory parameters by mechanistic domains

The IH group demonstrated a higher inflammatory burden, with elevated CRP levels (9.8 ± 6.7 vs 6.4 ± 4.9 mg/L; *t* = 2.90, *P* = .006) and a higher NLR (3.8 ± 1.6 vs 3.1 ± 1.3; *t* = 2.46, *P* = .017), while WBC counts were not significantly different (7.6 ± 1.9 vs 7.1 ± 1.7 × 10^9^/L; *P* = .151). Nutritional status appeared less favorable in the IH group, with lower serum albumin (36.2 ± 4.1 vs 38.0 ± 3.8 g/L; *t* = −2.41, *P* = .019), whereas total protein levels were comparable. The IH group also exhibited lower hemoglobin values (101 ± 12 vs 106 ± 11 g/L; *t* = −2.29, *P* = .025), with no significant differences in ferritin or transferrin saturation. In the coagulation-related profile, d-dimer was higher in the IH group (0.78 ± 0.40 vs 0.60 ± 0.32 mg/L FEU; *t* = 2.54, *P* = .014), whereas platelet counts were similar. Conventional uremia-related markers, including blood urea nitrogen and serum creatinine, showed no significant between-group differences. For mineral metabolism, serum phosphate was higher in the IH group (1.88 ± 0.36 vs 1.72 ± 0.33 mmol/L; *t* = 2.45, *P* = .018), while serum calcium levels were comparable; intact PTH and ALP demonstrated nonsignificant trends toward higher values in IH patients (both *P* > .05; Table 3).

**Table 3:** Laboratory indices between groups by mechanistic categories.

Variable	IH group (n = 38)	Control group (n = 126)	Test statistic	*P* value
Inflammation-related				
CRP, mg/L	9.8 ± 6.7	6.4 ± 4.9	*t* = 2.90	.006
WBC, ×10^9^/L	7.6 ± 1.9	7.1 ± 1.7	*t* = 1.46	.151
NLR	3.8 ± 1.6	3.1 ± 1.3	*t* = 2.46	.017
Nutrition/protein metabolism				
Albumin, g/L	36.2 ± 4.1	38.0 ± 3.8	*t* = −2.41	.019
Total protein, g/L	63.5 ± 5.8	64.2 ± 5.6	*t* = −0.66	.514
Anemia/iron metabolism				
Hemoglobin, g/L	101 ± 12	106 ± 11	*t* = −2.29	.025
Ferritin, ng/mL	320 ± 150	290 ± 140	*t* = 1.10	.277
Transferrin saturation, %	27.5 ± 8.6	28.8 ± 8.2	*t* = −0.83	.412
Coagulation/platelet-related				
Platelet count, ×10^9^/L	210 ± 58	220 ± 61	*t* = −0.92	.361
d-dimer, mg/L FEU	0.78 ± 0.40	0.60 ± 0.32	*t* = 2.54	.014
Uremia-related				
BUN, mmol/L	22.5 ± 5.2	21.8 ± 5.0	*t* = 0.73	.466
Serum creatinine, μmol/L	890 ± 180	870 ± 170	*t* = 0.61	.546
Mineral metabolism/vascular calcification-related				
Calcium, mmol/L	2.16 ± 0.15	2.18 ± 0.14	*t* = −0.73	.467
Phosphate, mmol/L	1.88 ± 0.36	1.72 ± 0.33	*t* = 2.45	.018
Intact PTH, pg/mL	420 ± 180	360 ± 160	*t* = 1.85	.070
ALP, U/L	112 ± 45	98 ± 41	*t* = 1.72	.092

ALP = alkaline phosphatase, BUN = blood urea nitrogen, CRP = C-reactive protein, FEU = fibrinogen equivalent units, IH = intimal hyperplasia, NLR = neutrophil-to-lymphocyte ratio, PTH = parathyroid hormone, WBC = white blood cell count.

### 3.4. Univariate analysis for screening potential risk factors of AVF intimal hyperplasia

In this cohort, longer hemodialysis vintage was associated with a higher likelihood of IH (OR 1.21 per 1-year increase, *P* = .021). Diabetes mellitus (OR 2.56, *P* = .013), peripheral artery disease/clinical indicators suggestive of vascular calcification (OR 3.26, *P* = .010), and recent infection within 6 months (OR 3.39, *P* = .010) were also positively associated with IH. With respect to treatment exposure, antiplatelet therapy showed a significant association with IH (OR 2.33, *P* = .030), whereas demographic factors including age, sex, and BMI were not associated with IH in univariate testing. Dialysis adequacy appeared to be inversely related to IH risk, with higher *Kt*/*V* associated with lower odds of IH (OR 0.79 per 0.1 increase, *P* = .012). Among vascular access-level variables, the duration from AVF creation to index evaluation demonstrated a strong positive association with IH (OR 1.16 per 1-month increase, *P* < .001). A history of access stenosis was also associated with IH (OR 2.80, *P* = .021), while a history of access thrombosis showed a borderline association (OR 2.53, *P* = .063). Laboratory indices suggested a mechanistic pattern involving inflammation, nutritional status, anemia, coagulation activation, and mineral metabolism. Higher CRP (OR 1.16 per 1 mg/L increase, *P* < .001), higher NLR (OR 1.51 per 1-unit increase, *P* = .003), higher d-dimer (OR 1.28 per 0.1 mg/L FEU increase, *P* < .001), and higher serum phosphate (OR 1.19 per 0.1 mmol/L increase, *P* = .004) were associated with increased IH risk. In contrast, higher albumin (OR 0.86 per 1 g/L increase, *P* = .003) and higher hemoglobin (OR 0.94 per 1 g/L increase, *P* < .001) were inversely associated with IH. Serum calcium showed a modest inverse association with IH (OR 0.75 per 0.1 mmol/L increase, *P* = .042). These variables with *P* < .10 were considered candidates for inclusion in the multivariable model (Table 4).

**Table 4:** Univariate logistic regression for screening potential risk factors of AVF intimal hyperplasia.

Variable	OR	95% CI	*P* value
Age (per 1 yr increase)	1.00	0.96–1.04	.920
Male sex (yes vs no)	1.09	0.52–2.31	.820
BMI (per 1 kg/m^2^ increase)	1.00	0.90–1.12	.964
Hemodialysis vintage (per 1 yr increase)	1.21	1.03–1.41	.021
Diabetes mellitus (yes vs no)	2.56	1.22–5.38	.013
Peripheral artery disease/vascular calcification signs (yes vs no)	3.26	1.33–7.97	.010
Recent infection within 6 mo (yes vs no)	3.39	1.33–8.65	.010
Antiplatelet therapy (yes vs no)	2.33	1.08–4.99	.030
*Kt*/*V* (per 0.1 increase)	0.79	0.66–0.95	.012
Time from AVF creation to evaluation (per 1 mo increase)	1.16	1.09–1.23	<.001
History of access thrombosis (yes vs no)	2.53	0.95–6.76	.063
History of access stenosis (yes vs no)	2.80	1.17–6.72	.021
CRP (per 1 mg/L increase)	1.16	1.07–1.24	<.001
NLR (per 1 unit increase)	1.51	1.15–1.98	.003
Albumin (per 1 g/L increase)	0.86	0.77–0.95	.003
Hemoglobin (per 1 g/L increase)	0.94	0.90–0.97	<.001
d-dimer (per 0.1 mg/L FEU increase)	1.28	1.12–1.46	<.001
Serum phosphate (per 0.1 mmol/L increase)	1.19	1.06–1.33	.004
Serum calcium (per 0.1 mmol/L increase)	0.75	0.57–0.99	.042

AVF = arteriovenous fistula, BMI = body mass index, CI = confidence interval, CRP = C-reactive protein, FEU = fibrinogen equivalent units, IH = intimal hyperplasia, NLR = neutrophil-to-lymphocyte ratio, OR = odds ratio.

### 3.5. Multivariable analysis of independent factors associated with AVF intimal hyperplasia

In the multivariable logistic regression model, a longer time from AVF creation to index evaluation remained independently associated with IH (aOR 1.10 per month, 95% CI: 1.04–1.17, *P* = .001). Diabetes mellitus was also an independent correlate of IH (aOR 2.08, 95% CI: 1.01–4.29, *P* = .047). Inflammatory burden retained significance after adjustment, with higher CRP associated with increased odds of IH (aOR 1.11 per 1 mg/L, 95% CI: 1.03–1.21, *P* = .008). Conversely, better nutritional status reflected by higher serum albumin was inversely associated with IH (aOR 0.90 per 1 g/L, 95% CI: 0.82–0.99, *P* = .028). Elevated serum phosphate remained an independent metabolic factor linked to IH (aOR 1.14 per 0.1 mmol/L, 95% CI: 1.02–1.28, *P* = .021). Age, hemodialysis vintage, *Kt*/*V*, and clinical indicators suggestive of peripheral artery disease/vascular calcification did not reach statistical significance in the fully adjusted model, although the direction of effect was consistent with the univariate findings (Table 5).

**Table 5:** Multivariable logistic regression for independent factors associated with AVF intimal hyperplasia.

Variable	Adjusted OR (aOR)	95% CI	*P* value
Age (per 1-yr increase)	1.01	0.97–1.06	.612
Hemodialysis vintage (per 1-yr increase)	1.12	0.95–1.33	.179
Diabetes mellitus (yes vs no)	2.08	1.01–4.29	.047
Peripheral artery disease/clinical signs of vascular calcification (yes vs no)	2.15	0.93–4.99	.073
*Kt*/*V* (per 0.1 increase)	0.88	0.74–1.04	.130
Time from AVF creation to evaluation (per 1-mo increase)	1.10	1.04–1.17	.001
CRP (per 1 mg/L increase)	1.11	1.03–1.21	.008
Albumin (per 1 g/L increase)	0.90	0.82–0.99	.028
Phosphate (per 0.1 mmol/L increase)	1.14	1.02–1.28	.021
d-dimer (per 0.1 mg/L FEU increase)	1.12	0.99–1.27	.071

aOR = adjusted odds ratio, AVF = arteriovenous fistula, CI = confidence interval, CRP = C-reactive protein, FEU = fibrinogen equivalent units, OR = odds ratio.

## 4. Discussion

This retrospective analysis evaluated clinical, dialysis-related, vascular access, and biochemical correlates of intimal hyperplasia (IH) in native arteriovenous fistulas (AVFs) among patients receiving maintenance hemodialysis. The principal findings were that a longer interval from AVF creation to index evaluation, diabetes mellitus, elevated systemic inflammatory burden measured by C-reactive protein (CRP), and higher serum phosphate were independently associated with IH, whereas higher serum albumin was inversely associated with IH. These results support a multifactorial conceptual model in which cumulative access exposure, metabolic disease, inflammation, nutritional status, and disordered mineral metabolism converge to promote maladaptive vascular remodeling. Contemporary mechanistic reviews emphasize that venous neointimal hyperplasia arises from complex interactions among endothelial injury, smooth muscle cell/fibroblast proliferation, extracellular matrix remodeling, macrophage polarization, and disturbed hemodynamics, providing biological plausibility for the observed clinical and biochemical associations.^[13,14]^

Although CRP and albumin remained independently associated with IH in multivariable analysis, these biomarkers should not necessarily be interpreted as direct causal mediators. Instead, they may partly represent co-occurring indicators of systemic inflammatory burden, malnutrition-inflammation complex status, or underlying disease severity. Furthermore, reverse causality is possible because progressive AVF dysfunction and IH-related hemodynamic abnormalities may themselves contribute to chronic inflammatory activation and nutritional deterioration. Therefore, potential surveillance bias cannot be excluded. Experimental and translational work has shown that disturbed flow in the juxta-anastomotic region correlates with endothelial loss, thrombus formation, and neointimal hyperplasia, underscoring the importance of hemodynamic forces in IH pathogenesis. Although AVF location and configuration were not significantly different between groups in our dataset, access duration appears to capture cumulative hemodynamic and inflammatory insults that are not fully reflected by anatomical categories alone. Diabetes mellitus emerged as an independent correlate of IH after adjustment. This aligns with clinical observations that diabetic patients experience higher risks of AVF maturation problems and long-term dysfunction, potentially mediated through endothelial dysfunction, oxidative stress, advanced glycation end products, and augmented pro-inflammatory signaling within the vascular wall.^[13,15]^ The present results extend these concepts by suggesting that diabetes may contribute not only to early maturation challenges but also to IH-related lesion development during ongoing maintenance use.

Systemic inflammation remained significant in the multivariable model, with higher CRP associated with increased odds of IH and albumin exerting a protective association. This pattern is consistent with the emerging clinical emphasis on composite inflammatory-nutritional phenotypes as determinants of AVF outcomes. A large retrospective cohort has demonstrated that an elevated CRP-to-albumin ratio is associated with subsequent AVF dysfunction, supporting the concept that chronic low-grade inflammation coupled with impaired nutritional reserve predisposes to adverse access remodeling.^[16]^ Our findings similarly suggest that, even when evaluated as individual markers, higher CRP and lower albumin identify a biological milieu favoring neointimal proliferation and reduced vascular resilience. Serum phosphate was independently associated with IH, indicating that disordered mineral metabolism may be relevant to vascular access remodeling. Recent clinical evidence indicates that higher phosphorus levels are linked to AVF dysfunction in hemodialysis cohorts.^[8,17]^ Mechanistically, hyperphosphatemia may promote vascular smooth muscle cell osteogenic transition, vascular stiffness, endothelial injury, and pro-inflammatory macrophage activity, thereby facilitating stenotic lesion formation within AVF outflow segments.^[18]^ Similarly, elevated phosphate should be interpreted as a clinically associated metabolic marker rather than definitive proof of causality in IH formation within the limitations of this retrospective study design.

The absence of a definitive independent association of PTH and alkaline phosphatase in our multivariable model may reflect limited sample size, the cross-sectional biochemical snapshot, and heterogeneity in CKD–MBD medication exposure. Several associations observed in univariate analyses were attenuated after adjustment. Hemodialysis vintage and dialysis adequacy (*Kt*/*V*) may be partially mediated through inflammation and nutritional status or may correlate with access duration, suggesting interdependence among these predictors. Similarly, d-dimer and clinical indicators suggestive of peripheral artery disease or vascular calcification showed directional consistency but did not reach statistical significance in the adjusted model. Given the pathophysiologic links between thrombosis, calcific vasculopathy, and neointimal remodeling, these variables may still merit consideration in larger datasets with more granular imaging and longitudinal surveillance.

Recent studies and contemporary reviews provide a convergent framework that supports the main signals observed in this analysis. Inflammation has been increasingly recognized as a determinant of AVF dysfunction and stenosis. A 2023 retrospective study in a Chinese hemodialysis cohort reported that the preoperative CRP-to-albumin ratio significantly predicted AVF dysfunction, emphasizing the interplay between systemic inflammatory stress and nutritional reserve in access outcomes.^[16]^ In parallel, prospective and model-development studies using immune-inflammation indices have reinforced the prognostic relevance of systemic inflammatory burden for vascular access failure, consistent with our finding that CRP remained independently associated with IH.^[19]^ Mineral metabolism has also emerged as an increasingly investigated axis. A 2023 study identified phosphorus level as an independent risk factor for AVF dysfunction, suggesting that hyperphosphatemia may contribute to failure trajectories beyond classic cardiovascular endpoints.^[17]^ More recently, a 2025 investigation further supported hyperphosphatemia as an independent predictor of AVF dysfunction, strengthening the clinical rationale for phosphate control as a potentially modifiable contributor to access longevity.^[8]^ Our data extend these observations by linking higher phosphate to IH specifically, thereby complementing broader dysfunction-focused endpoints.

Hemodynamic mechanisms remain central to current conceptual models. Experimental work published in 2024 demonstrated that disturbed flow in the juxta-anastomotic region correlates with endothelial loss and neointimal hyperplasia, lending mechanistic support to our finding that longer AVF exposure time is a strong correlate of IH.^[14]^ A 2024 mechanistic review further summarized the roles of macrophage activation, extracellular matrix remodeling, and lipid/mineral metabolic perturbations in IH development, providing a plausible biological substrate for the combined signals of diabetes, inflammation, and phosphate in our multivariable model.^[13]^ With respect to metabolic disease, reviews published in 2024 have highlighted that elderly diabetic patients face higher risks of AVF immaturity and suboptimal outcomes.^[15]^ Although these analyses often focus on early maturation, the pathobiology of diabetes-related endothelial dysfunction and heightened inflammatory tone is likely relevant to longer-term IH susceptibility, consistent with our adjusted association for diabetes. Taken together, the contemporary literature suggests that IH is driven by intersecting clinical and biochemical domains – metabolic disease, inflammation-nutrition imbalance, mineral dysregulation, and hemodynamic injury – rather than a single dominant factor. Our findings are aligned with this multi-domain view and provide additional evidence that routinely available clinical variables may capture these mechanistic pathways in real-world dialysis populations.

The present findings support targeted, risk-stratified surveillance for IH in hemodialysis patients with diabetes, elevated CRP, lower albumin, and higher phosphate, particularly when AVFs have been in use for longer periods. In routine practice, integrating these variables into structured follow-up may help identify patients who warrant earlier duplex ultrasound assessment or proactive access-preservation strategies. The independent association of phosphate with IH also reinforces the importance of optimizing CKD–MBD management and adherence to phosphate-lowering interventions as part of comprehensive vascular access care.^[20,21]^

Several limitations should be noted. First, the retrospective and single-center design limits causal inference and may introduce selection bias. Second, IH ascertainment may be influenced by the timing and indications for imaging, and the absence of standardized longitudinal ultrasound schedules could lead to under-detection of subclinical lesions. Third, laboratory variables were analyzed as single time-point measures; dynamic trajectories of CRP, albumin, and phosphate may better reflect cumulative biological exposure. Fourth, residual confounding is possible, including unmeasured factors such as detailed cannulation practices, access flow measurements, medication dose intensity, and inflammatory or oxidative biomarkers beyond standard panels. Finally, the relatively small IH event count may restrict the stability of multivariable estimates and limit exploration of interaction effects (e.g., diabetes-by-phosphate or inflammation-by-nutrition). Future multicenter, prospective studies with standardized imaging protocols and time-updated biochemical models are warranted to refine risk stratification and validate the observed associations. In particular, the observed association between AVF exposure duration and IH may partly reflect surveillance intensity and cumulative follow-up opportunity in addition to biological remodeling processes. Because DUS surveillance was not systematically protocolized for all patients, ascertainment bias and under-detection of subclinical IH lesions remain possible.

## 5. Conclusion

In this retrospective cohort of hemodialysis patients with native AVFs, intimal hyperplasia was independently associated with a longer time from AVF creation to evaluation, diabetes mellitus, higher CRP, and elevated serum phosphate, whereas higher albumin was protective. These findings suggest that cumulative access exposure, metabolic disease, inflammatory-nutritional status, and disordered mineral metabolism are clinically associated with IH in hemodialysis AVFs and may help inform risk-stratified surveillance and preventive management strategies.

## Author contributions

**Conceptualization:** Mingli Liang, Xiaoli Liao.

**Data curation:** Mingli Liang, Xiaoli Liao.

**Formal analysis:** Mingli Liang, Xiaoli Liao.

**Funding acquisition:** Mingli Liang, Xiaoli Liao.

**Investigation:** Xiaoli Liao.

**Writing – original draft:** Xiaoli Liao.

**Writing – review & editing:** Xiaoli Liao.

## References

[1] Lok CE Yuo T Lee T. Hemodialysis vascular access: core curriculum 2025. Am J Kidney Dis. 2025;85:236–52. 39625430 10.1053/j.ajkd.2024.05.021

[2] Cao Y Guo F Chen D Li L Jie X Wang W. Exosomes in arteriovenous fistula stenosis. Front Cell Dev Biol. 2025;13:1663973. 41049292 10.3389/fcell.2025.1663973 PMC12492956

[3] Shiu Y-T Northrup H Huang Y Cho ME Bunsawat K. Cellular and molecular mechanisms underlying hemodialysis arteriovenous fistula dysfunction and approaches to promote maturation: a vascular perspective. Am J Physiol Heart Circ Physiol. 2025;329:H241–57. 40465509 10.1152/ajpheart.00010.2025 PMC12237592

[4] Zhao M Wu Q Zhao Y Nian R Li W Lu H. Tissue metabolomics reveals metabolic dysregulation associated with intimal hyperplasia in arteriovenous fistula stenosis. Front Physiol. 2025;16:1638179. 40901611 10.3389/fphys.2025.1638179 PMC12399881

[5] Shu P Huang L Huo S. Machine learning-based risk prediction model for arteriovenous fistula stenosis. Eur J Med Res. 2025;30:217. 40156016 10.1186/s40001-025-02490-x PMC11954292

[6] Fu M Liu F Ren C. Bioinformatics and systems biology to identify underlying common pathogenesis of diabetic kidney disease and stenosis of arteriovenous fistula. BMC Nephrol. 2025;26:299. 40597829 10.1186/s12882-025-04239-4 PMC12211224

[7] Zhao B Fu G Zhan S. Development and validation of an inflammatory-based nomogram for predicting arteriovenous fistula maturation failure in end-stage renal disease patients. Langenbecks Arch Surg. 2025;410:231. 40719921 10.1007/s00423-025-03819-0 PMC12304049

[8] Jiang Y Cui C Nan H Chang T Li F Zhang S. Hyperphosphatemia as a potential risk factor for arteriovenous fistula dysfunction: a retrospective study in hemodialysis patients. PLoS One. 2025;20:e0335599. 41166265 10.1371/journal.pone.0335599 PMC12574839

[9] Granata A Maccarrone R Spatola L; Integrated Imaging and Interventional Nephrology Working Group of the Italian Society of Nephrology (SIN) and of the Italian Society for Ultrasound in Medicine and Biology (SIUMB). A novel duplex ultrasound-based classification of outflow stenosis in native arterio-venous fistulas for hemodialysis. BMC Nephrol. 2025;26:670. 41299362 10.1186/s12882-025-04595-1 PMC12659217

[10] Lawrie K O’Neill S Malik J. Classifications of haemodialysis vascular access stenosis: a scoping review. BMJ Open. 2025;15:e088045. 10.1136/bmjopen-2024-088045 PMC11751806 39819957

[11] Pichot O Diard A Bosc JY.; AV Access Network Investigators. Standardized methodology for duplex ultrasound examination of arteriovenous access for hemodialysis: a proposal of the French Society of Vascular Medicine and the French-Speaking Society of Vascular Access. Ultrasound Med Biol. 2023;49:2213–20. 37544830 10.1016/j.ultrasmedbio.2023.07.007

[12] Wo K Morrison BJ Harada RN. Developing duplex ultrasound criteria for diagnosis of arteriovenous fistula stenosis. Ann Vasc Surg. 2017;38:99–104. 27521824 10.1016/j.avsg.2016.04.013

[13] Yan R Song A Zhang C. The pathological mechanisms and therapeutic molecular targets in arteriovenous fistula dysfunction. Int J Mol Sci. 2024;25:9519. 39273465 10.3390/ijms25179519 PMC11395150

[14] Bai H Varsanik MA Thaxton C. Disturbed flow in the juxta-anastomotic area of an arteriovenous fistula correlates with endothelial loss, acute thrombus formation, and neointimal hyperplasia. Am J Physiol Heart Circ Physiol. 2024;326:H1446–61. 38578237 10.1152/ajpheart.00054.2024 PMC11380968

[15] Liu S Wang Y He X Wang Y Li X. Factors affecting suboptimal maturation of autogenous arteriovenous fistula in elderly patients with diabetes: a narrative review. Heliyon. 2024;10:e35766. 39170451 10.1016/j.heliyon.2024.e35766 PMC11337043

[16] Hu S Wang R Ma T. Association between preoperative C-reactive protein to albumin ratio and late arteriovenous fistula dysfunction in hemodialysis patients: a cohort study. Sci Rep. 2023;13:11184. 37433824 10.1038/s41598-023-38202-w PMC10336133

[17] Zhang F Li J Yu J. Risk factors for arteriovenous fistula dysfunction in hemodialysis patients: a retrospective study. Sci Rep. 2023;13:21325. 38044365 10.1038/s41598-023-48691-4 PMC10694134

[18] Fusaro M Barbuto S Gallieni M. Real-world usage of chronic kidney disease - mineral bone disorder (CKD-MBD) biomarkers in nephrology practices. Clin Kidney J. 2024;17:sfad290. 38223338 10.1093/ckj/sfad290 PMC10784916

[19] Ren S Xv C Wang D. The predictive value of systemic immune-inflammation index for vascular access survival in chronic hemodialysis patients. Front Immunol. 2024;15:1382970. 38827733 10.3389/fimmu.2024.1382970 PMC11140091

[20] Bai H Li Z Zhang W. Early thrombus formation is required for eccentric and heterogeneous neointimal hyperplasia under disturbed flow. J Thromb Haemost. 2024;22:3614–28. 39173878 10.1016/j.jtha.2024.07.028 PMC11608155

[21] Xu Y Korayem A Cruz-Solbes AS. Inhibition of endothelial-to-mesenchymal transition in a large animal preclinical arteriovenous fistula model leads to improved remodelling and reduced stenosis. Cardiovasc Res. 2024;120:1768–79. 39056563 10.1093/cvr/cvae157 PMC11587554

